# Pre-clinical evaluation of a potent and effective Pin1-degrading agent in pancreatic cancer

**DOI:** 10.1016/j.omton.2025.201078

**Published:** 2025-11-01

**Authors:** Giulia Alboreggia, Tiane Li, Anne Marie Prentiss, Parima Udompholkul, Frank Xia, Tim Synold, Jun Wu, Mingye Feng, Mustafa Raoof, Maurizio Pellecchia

**Affiliations:** 1 Division of Biomedical Sciences, School of Medicine, University of California, Riverside, 900 University Avenue, Riverside, CA 92521, USA; 2 Department of Surgery, City of Hope, Duarte, CA 91010, USA; 3 Analytical Pharmacology Core, City of Hope, Duarte, CA 91010, USA; 4 Animal Tumor Model Core, City of Hope, Duarte, CA 91010, USA; 5 Department of Immuno-Oncology, City of Hope, Duarte, CA 91010, USA

**Keywords:** MT: Regular Issue, Pin1, molecular crowbars, cancer-associated fibroblasts, PDAC

## Abstract

The peptidyl-prolyl isomerase protein Pin1 has been shown to contribute to cancer onset, development, and progression by regulating the function and stability of oncogenes and tumor suppressors, both in cancer cells and surrounding stromal cells. Hence, it has long been sought as a possible therapeutic target in oncology. However, further validation of Pin1 as a suitable target for novel therapeutic strategies requires the development of potent and effective pharmacological inhibitors, which are currently scarce. Very recently, we reported novel covalent Pin1 inhibitors that, acting as molecular crowbars, destabilize the protein *in vitro*, causing its degradation in cells. Here, we advanced those agents by improving their plasma stability. We further characterized their mode of action on cancer cells and cancer-associated fibroblasts and studied their efficacy in a syngeneic mouse model of pancreatic cancer peritoneal metastases. Our studies support the hypothesis that these novel Pin1 degraders could be translated into effective therapeutics against peritoneal metastases in pancreatic and other types of gastrointestinal and abdominal cancers that develop peritoneal metastases.

## Graphical abstract

**Figure:**
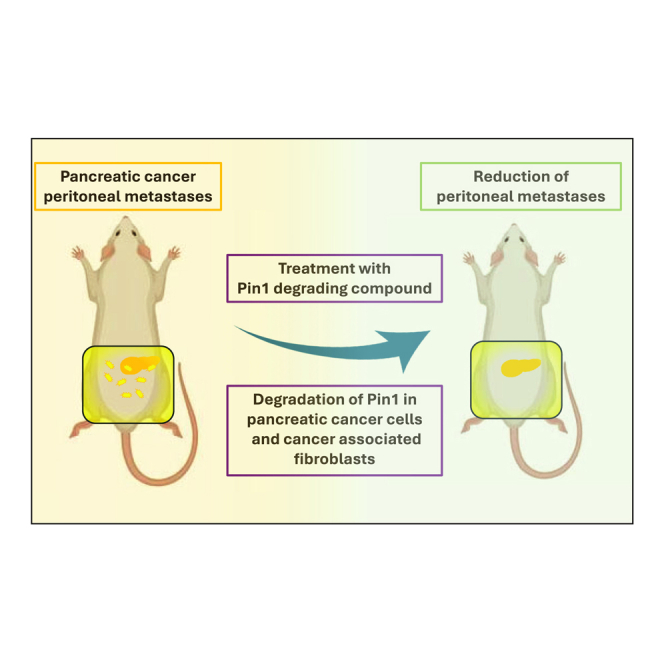

## Teaser

Pin1 regulates the function of oncogenes and tumor suppressors, both in cancer cells and surrounding stromal cells. Novel Pin1-degrading compounds were tested in a syngeneic mouse model of pancreatic cancer peritoneal metastases. The study suggests that Pin1 degraders could be translated into therapeutics against tumors that develop peritoneal metastases.

## Introduction

The enzyme Pin1 is a peptidyl-prolyl cis/trans isomerase that binds to phosphorylated serine/threonine-proline (pSer/Thr-Pro) motifs, inducing peptide bond isomerization. Pin1 has been shown to play a major role in modulating protein function, and its overexpression is implicated in the onset and progression of several cancers,^1^^,^^2^^,^^3^^,^^4^ activating several oncoproteins, such as those in the KRAS pathway,^5^ while simultaneously inactivating tumor suppressors.^1^^,^^4^ Overexpression of Pin1 in patients with cancer directly correlates with lymph node metastasis in those with non-small cell lung cancer, rapid disease progression in those with oral squamous carcinoma, and overall poor clinical outcomes.^6^^,^^7^^,^^8^^,^^9^ In addition, genetically engineered mice lacking Pin1 are highly resistant to tumorigenesis and are phenotypically normal,^4^^,^^10^^,^^11^^,^^12^ while in humans, genetic alterations that result in diminished Pin1 expression correlate with a reduced incidence of cancer.^13^ Activation of the KRAS pathway by Pin1 is particularly important for the development of pancreatic ductal adenocarcinoma (PDAC), one of the most aggressive and lethal tumors, which is projected to become the second leading cause of cancer-related deaths by 2030 in the United States.^14^ PDAC is particularly difficult to treat given its resistance to most therapeutic strategies, including chemotherapy, targeted therapies, and even immunotherapy.^15^^,^^16^ This treatment resistance is most commonly attributable to the PDAC tumor microenvironment that is highly desmoplastic and immunosuppressive.^17^^,^^18^^,^^19^ Pin1 overexpression induces chromosome instability and tumorigenesis in PDAC, and it is highly expressed both in tumor cells and cancer-associated fibroblasts (CAFs).^20^^,^^21^ Based on these premises, potential therapeutic targeting for PDAC includes inhibition of Pin1 in tumor cells and in CAFs.^21^

In recent years, several attempts have been made to develop novel, potent, and selective Pin1 inhibitors, including small-molecule inhibitors,^22^^,^^23^^,^^24^^,^^25^ peptide mimetics,^26^^,^^27^^,^^28^ covalent agents targeting the active-site Cys113,^29^^,^^30^ and even a proteolysis-targeted chimera (PROTAC) agent that can more effectively cause Pin1 degradation in cells.^31^ Most recently, however, we reported a novel class of Pin1 degraders that bind covalently to Cys113 and are designed to induce Pin1 destabilization *in vitro* and in cells.^32^ As detailed in our recent publication,^32^ our strategy for developing these novel agents was based on iterative structure-activity relationship studies, in which the binding affinity of synthetic compounds for Pin1 was monitored via a biochemical assay, while each compound was simultaneously tested for its ability to induce thermal destabilization of Pin1 *in vitro*.^32^ Using this innovative strategy, we discovered two agents that bound covalently to Pin1, caused its thermal instability *in vitro*, and resulted in Pin1 degradation in cells.^32^ Based on structural studies detailing the molecular interactions between these agents and Pin1—using nuclear magnetic resonance (NMR) spectroscopy and X-ray crystallography—the agents appeared to act as molecular crowbars, causing conformational instability in Pin1 that exposed hydrophobic residues, including Trp73, which in cells triggered ubiquitin-independent degradation.^32^ Here, we further assessed the pharmacological properties of these agents, introduced additional chemical modifications that improved their plasma stability, and conducted *in vivo* efficacy studies using a murine model of pancreatic cancer. Our findings suggest that these Pin1 degraders could be developed into novel and effective treatments for PDAC and other gastrointestinal and abdominal cancers.

## Results

### Covalent Pin1-destabilizing agents: Molecular crowbars causing Pin1 degradation in cells

**Figure 1:**
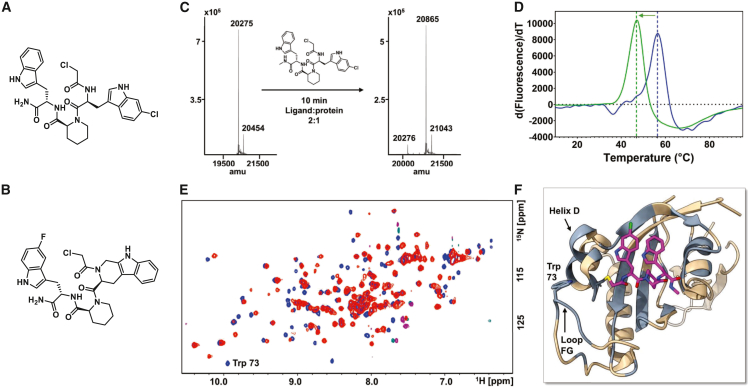
Structural characterizations of 164B8 binding to Pin1 (A and B) show the chemical structures of compounds 158H9 and 164A10, respectively, while the chemical structure of compound 164B8 is shown in (C). (C) presents mass spectrometry data revealing complex formation between Pin1 (MW 20275 and 20454 after phosphogluconosylation of the target in *E. coli* [+178]) and 164B8 [MW 625], resulting in covalent adducts of mass 20865 and 21043 (+178) (expected mass for the covalent adduct is 20864). (D) Thermal denaturation of Pin1 in the absence (blue) or presence (green) of 164B8, measured as described in the manuscript. The compound induces a thermal destabilization (ΔTm) of −9.55 ± 0.19°C (green arrow). Curves are normalized on the *y* axis for better illustration. (E) Superimposition of 2D [^15^N, ^1^H] correlation spectra for ^15^N-Pin1 (50 μM in buffer containing 50 mM phosphate, pH 7.5, 150 mM NaCl, 10% D_2_O, 1% DMSO) acquired in the absence (blue) or presence (red) of 250 μM 164B8 (after 3 h pre-incubation). The side-chain resonance for Trp73 is indicated. Full resonance assignments are reported in Figure S4. (F) Mapping of the chemical shifts induced by 164B8 into a model of the three-dimensional structure of the complex between Pin1 (ribbons, in gray are regions for which chemical shift perturbations are recorded upon complex formation with 164B8) and 164B8 (magenta stick model; nitrogen atoms are in blue, the chlorine atom is in green, and the sulfur atom of Pin1 Cys113 is in yellow) reveals regions affected by the binding, which include the binding site and loop FG and helix D. The model was prepared based on the X-ray structure with a similar agent as we recently reported.^32^

To assess the ability of our agents to bind and inhibit Pin1, we developed a robust biochemical assay based on the dissociation-enhanced lanthanide fluorescent immunoassay (DELFIA) platform. In our implementation, we used a biotinylated form of a known Pin1-binding peptide (D-peptide) to monitor the ability of test agents to inhibit the interaction between Pin1 and the D-peptide at various incubation times, as described previously.^32^ Hence, each new synthetic agent’s binding affinity was assessed by its ability to compete with the D-peptide in a displacement assay at a given incubation time (see also Figure S1, which compares the structure and properties of the D-peptide with one of our agents). In addition, as mentioned above and reported recently, we tested the ability of the agents to reduce Pin1 thermal stability *in vitro* by thermal denaturation measurements.^32^ In these characterizations, Pin1 was incubated with our covalent test agents in the presence of a fluorescent dye (SYPRO Orange), and the temperature of the samples was gradually increased in a linear gradient from 10°C to 95°C to induce thermal denaturation of Pin1. Pin1 unfolding causes a nonspecific interaction between the water-quenched fluorescent dye and the protein’s exposed hydrophobic residues, resulting in increased fluorescence. A shift in the thermal denaturation temperature between the free and bound protein is interpreted as a stabilization or destabilization event induced by the test ligand. Using these approaches, we initially derived agents **158H9** and **164A10** (Figures 1A and 1B), which showed IC_50_ values in the DELFIA assay of 21.5 ± 0.6 nM and 4.1 ± 0.2 nM, respectively (after 6 h preincubation), and caused negative denaturation shifts (ΔTm) of −10.01 ± 0.04°C and −9.63 ± 0.08°C, respectively (Table 1).^32^ To further assess whether these agents could be suitable for *in vivo* efficacy studies, we also performed stability assays in aqueous buffer and in plasma from various species (Table S2, Figure S2**)**. We tested the stability of our agents in plasma from multiple mouse strains as well as in human plasma (Figure S2), given the peptide nature of the compounds. For example, plasma from esterase-deficient (Es1-deficient) mice was included to determine the effect of circulating esterases known to be present in wildtype and B6-Cg mouse plasma. These studies revealed that although the agents were very stable in buffer, they underwent rapid degradation in mouse plasma, likely because of their peptide-like nature. Peptidases and esterases are particularly effective on linear peptide sequences with unmodified amino and carboxy termini; consequently, peptide stability can be improved through chemical modification of the termini, including N-methylation.^33^ Because the N-terminus of agents **158H9** and **164A10** is already modified (Figures 1A and 1B) with a chloroacetamide group that reacts with the Pin1 active-site residue Cys113, we introduced N-methylation of the terminal carboxyamide, yielding agent **164B8** (Figures 1C and S3 shows the synthetic scheme, characterization, and plasma stability studies for **164B8**).

**Table 1:** Chemical structures and characterizations of Pin1 covalent inhibitors

Cmpd	MW	IC_50_ (nM)	ΔT_m_ (°C)	TPSA (Å^2^)	% Complex (MS)
**158H9**	**610**	**21.5 ± 0.6**	**−10.01 ± 0.04**	**145.66**	**∼100%**
**164A10**	**606**	**4.1 ± 0.2**	**−9.63 ± 0.08**	**136.87**	**∼100%**
**164B8**	**625**	**4.65 ± 0.04**	**−9.55 ± 0.19**	**131.67**	**∼100%**

IC_50_ values were obtained by dose-response curves in a dissociation-enhanced lanthanide fluorescent immunoassay (DELFIA) displacement assay after a compound-ligand incubation time of 6 h, as described previously. Standard errors reported are from duplicate measurements. Topological polar surface areas (TPSA) were computed using ChemDraw 23.1.2. ΔTm values were measured in quadruplicates, as described in the manuscript. The percentage of complex formation was determined by mass spectrometry after incubation of ligand (20 μM) and Pin1 at a 2:1 ligand-to-protein ratio for 10 min (Figure 1). The chemical structures of the three compounds are reported in Figure 1.

Inclusion of the methyl group could also improve cell permeability by increasing compound lipophilicity, as reflected by a lower topological polar surface area (Table 1). Similar to the parent molecules, **164B8** bound covalently to Pin1 (Figure 1C) and induced substantial thermal destabilization of Pin1, with a ΔTm value of −9.55 ± 0.19°C (Table 1; Figure 1D). Interestingly, in the DELFIA assay, **164B8** was more potent than its parent molecule, **158H9** (Table 1). As anticipated, however, **164B8** was significantly more stable in all plasma samples tested (Table S2; Figure S3), making it a more suitable candidate for *in vivo* efficacy studies.

To further characterize the binding of **164B8** to Pin1, we studied its complex using NMR spectroscopy using a uniformly ^15^N-labeled sample of Pin1 (Figure 1E). We previously observed that binding of compounds in this class induced conformational changes in helix D and loop FG (Figure 1F), as evidenced by X-ray crystallography and ligand-induced chemical shift mapping of both Pin1 backbone ^15^N/^1^H resonances and the side chain ^1^H^ε^/^15^N^ε^ resonances of Trp73 (Figures 1E and 1F).^32^ We reported that the Trp73 side-chain resonances were substantially perturbed or broadened beyond detection by agents that caused Pin1 degradation in cells.^32^ Analysis of chemical shift changes induced by **164B8** in ^15^N-Pin1 likewise revealed perturbations that affected residues within the protein binding site and extended to loop FG and helix D, including broadening of the Trp73 side-chain resonance (Figures 1E and 1F). These findings suggest that **164B8** behaves similarly to the previously studies compounds, acting as a molecular crowbar that destablilizes the packing of loop FG and helix D with the rest of the protein domain.

**Figure 2:**
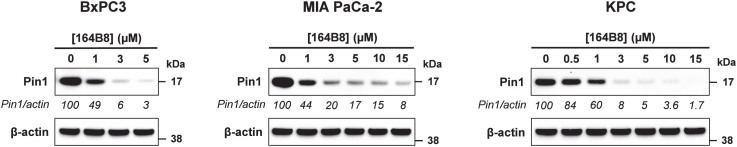
Degradation of Pin1 in different cell lines in response to 164B8 treatment BxPC3, MIA PaCa-2, and KPC were each treated with 0.1% DMSO and concentrations of 164B8 ranging between 0.5 and 15 μM for 24 h, and Pin1 levels were quantified with respect to β-actin using a specific anti-Pin1 antibody and western blotting (uncropped blots are reported in Figure S6).

To ascertain that the plasma-stable agent **164B8** retained its ability to induce Pin1 degradation in cells, we tested it in three pancreatic cancer cell lines—human BxPC3 and MIA PaCa-2, and the mouse pancreatic cancer cell line KPC—using western blot analysis (Figure 2).

**Figure 3:**
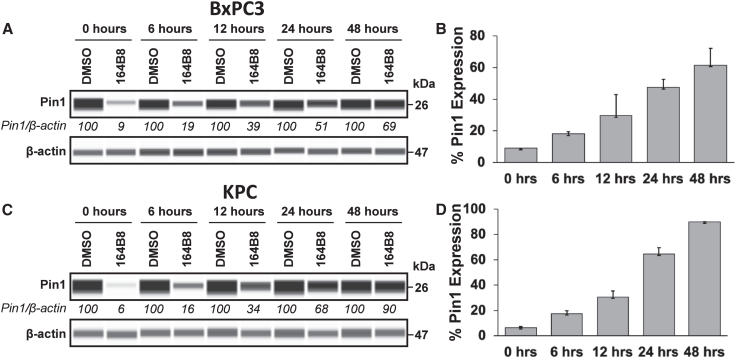
Western blot-like images of Pin1 recovery after degradation by 164B8 using capillary electrophoresis immunoassay (A) BxPC3 cells were pretreated with either 0.1% DMSO or 5 μM 164B8 for 24 h before washing and incubating in serum-free media to evaluate Pin1 recovery at 0, 6, 12, 24, and 48 h. (B) The experiment was performed in two independent replicates, and the results are plotted with the associated standard deviation. (C and D), as in (A) and (B), respectively, but testing the agents against the KPC cell line. Replicate experiments and full images are reported in Figure S7.

The agent effectively caused Pin1 degradation in all three cell lines, with sub-micromolar DC_50_ values comparable to those previously observed for **158H9** and **164A10**. To preliminarily assess the duration of Pin1 degradation in cancer cells, BxPC3 and KPC cells were treated with **164B8** for 24 h, washed, and then incubated in serum-free medium for various time points. Pin1 levels were then measured by capillary electrophoresis (Figure 3). These studies revealed that, after exposure to **164B8**, Pin1 levels were restored to approximately 50% after 48 h (Figure 3).

**Figure 4:**
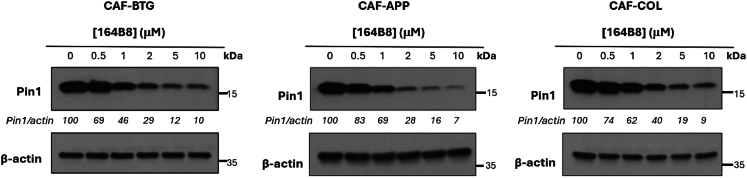
Pin1 degradation induced by 164B8 in primary CAFs CAFs isolated from patient-derived biliary tract (cholangiocarcinoma; CAF-BTG), appendiceal (CAF-APP), and colorectal cancer peritoneal metastasis tissues (CAF-COL) were treated with either 0.1% DMSO or the Pin1 inhibitor 164B8 (0.5–10 μM) for 24 h. Pin1 protein levels were assessed by immunoblotting using a specific anti-Pin1 antibody, with β-actin serving as the loading control. Uncropped blots are reported in Figure S8.

Pin1 is not only overexpressed in cancer cells but also markedly elevated in CAFs, where its expression correlates with desmoplastic features and poor clinical outcomes.^21^ These findings highlight the importance of targeting Pin1 not only in tumor cells but also within the stromal compartment. To evaluate whether our Pin1 inhibitor, **164B8**, can effectively target CAFs, we established primary normal fibroblast (NF) cultures from human peritoneum and primary CAF cultures from peritoneal metastases obtained from three individuals living with gastrointestinal cancer, including pancreaticobiliary, appendiceal, and colorectal cancer peritoneal metastases. Peritoneal metastases exhibit dense stromal architecture, in which CAFs play essential pro-tumorigenic functions; thus, targeting Pin1 in CAFs may have direct treatment relevance.^21^ We observed that Pin1 expression was increased in CAFs compared with NFs (Figure S9). Furthermore, treatment with **164B8** induced a dose-dependent degradation of Pin1 across all CAF types, demonstrating its broad efficacy against CAFs derived from individuals with diverse tumor origins **(**Figure 4**).**

Taken together, these studies identify **164B8** as a potent, targeted Pin1 agent with improved pharmacokinetic properties compared with the parent molecules, while maintaining its Pin1-degrading effect in both cancer cells and CAFs. These findings provide a framework for evaluating the efficacy of **164B8** in mouse models of cancer.

### Pin1 degrader **164B8** causes Pin1 degradation *in vivo* and suppresses tumor metastases

The effect of Pin1 degradation by **164B8** on cell viability was assessed using live-cell analysis (IncuCyte S3). The resulting EC_50_ values after 72 h were 8.4 μM in MIA PaCa-2 cells and 5.3 μM in KPC cell lines, respectively (Figure S11). Furthermore, to evaluate the acute single-dose toxicity of **164B8** *in vivo*, CD-1 immune-competent mice were exposed to increasing doses of **164B8**. The highest soluble dose of 100 mg/kg (administered intraperitoneally) did not produce any observable signs of acute toxicity when the mice were monitored over a 2-week period. Therefore, a maximum tolerated dose was not reached (Figure S12).

**Figure 5:**
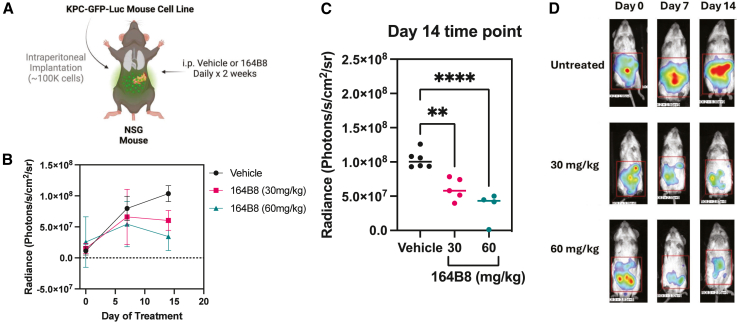
*In vivo* efficacy studies with 164B8 in a murine model of metastatic pancreatic cancer 164B8 caused a significant, dose-dependent reduction in peritoneal tumor burden compared to the vehicle control. (A) The mouse model used involved i.p injection of KPC-GFP-Luc pancreatic cancer cells in NSG mice. (B) Tumor burden in different treatment cohorts (*n* = 4–6 per group) was determined weekly using whole-body imaging. (C) Statistical significance of the reduction in tumor burden in each cohort (ANOVA with Dunnett’s multiple comparison test: vehicle vs. 30 mg/kg, *p* value = 0.0021; vehicle vs. 60 mg/Kg *p* value < 0.001). (D) Representative luminescence whole-body images of mice from each of the three groups taken on days 0, 7, and 14. The complete dataset and images are reported in Figure S14.

To test the efficacy and toxicity of **164B8** in a murine model of metastatic pancreatic cancer, GFP- and luciferase-expressing KPC pancreatic cancer cells (100,000 cells in 100 μL; Figure 5A) were injected into the peritoneal cavity of NSG mice (*n* = 4–6 per group). 72 h after the establishment of peritoneal metastases, the mice were randomized, and each cohort received either the vehicle alone (control), 30 mg/kg, or 60 mg/kg of the study agent daily for 2 weeks. Luminescence imaging was performed at baseline and weekly thereafter to determine cancer growth and peritoneal metastasis burden (Figures 5B, 5C, and 5D). Both doses resulted in a significant, dose-dependent reduction in tumor burden (as measured by luminescence) compared with the untreated cohort (Figure 5).

**Figure 6:**
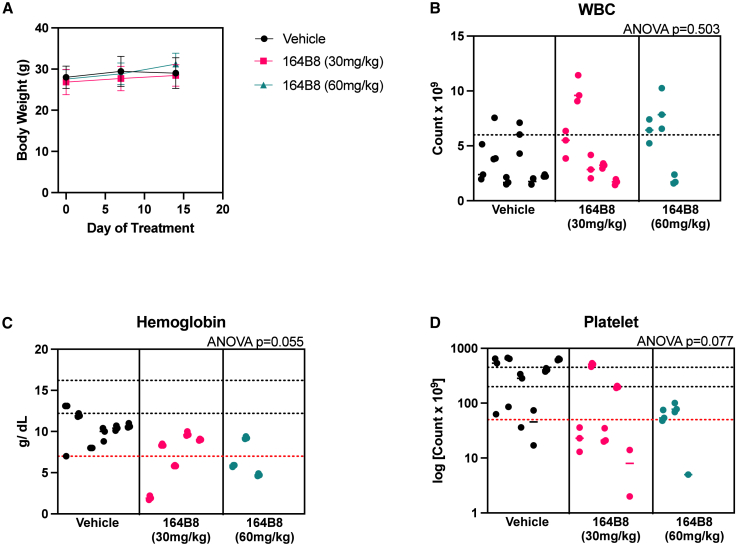
Toxicity of 164B8 after repeated doses in NSG mice Blood from mice treated as described in Figure 5 was collected and evaluated at the end of the experiment. Peripheral blood from mice was collected and analyzed by standard complete blood count (CBC). (A) Body weight, (B) white blood cell (WBC) counts (×10^9^ cells/L), (C) hemoglobin (HGB) concentration (g/dL), and (D) platelet counts (×10^9^ cells/L, log scale) in peripheral blood were determined. Data are presented as individual values with mean ± SD. Dotted horizontal lines indicate physiological reference ranges. Statistical comparisons were performed using one-way AVOVA; *p* values are shown in each panel. Based on CBC counts, 164B8 caused mild anemia compared to control and moderate thrombocytopenia, possibly indicating on-target side effects of Pin1 inhibition.

Treatment with **164B8** was generally well tolerated at 30 mg/kg, with mice presenting only mild anemia and moderate thrombocytopenia (Figures 6 and S13). At the final time point, two of six mice in the high-dose group (60 mg/kg) were euthanized because of ascites development, although no obvious carcinomatosis was observed.

**Figure 7:**
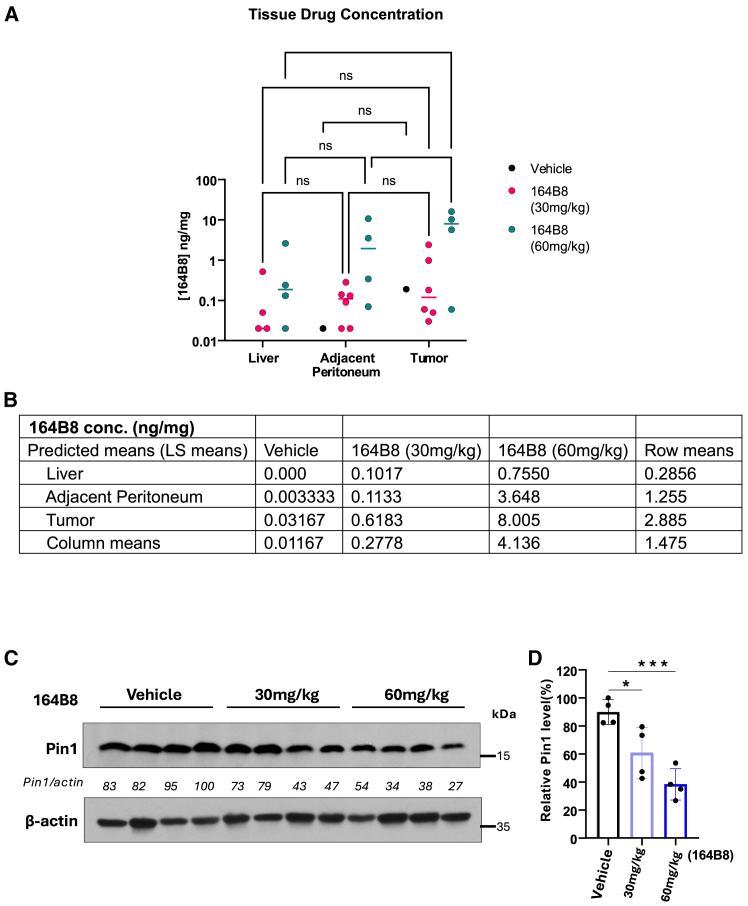
Determination of Pin1 levels and 164B8 concentration in treated mice Pin1 drug concentration and Pin1 levels in tumors were measured 6 h after the last dose of 164B8 treatment by LC-MS (A and B) and western blot (C), respectively. Drug concentrations among the three groups were compared using two-way ANOVA in Prism, accounting for interactions (D). Tukey’s multiple comparison test was used to compare multiple groups (∗∗∗*p* = 0.0003, ∗*p* = 0.035). Pin1 levels were compared among the three groups after normalizing to β-actin (loading control) using one-way ANOVA and adjusting for multiple comparisons (∗∗∗*p* < 0.001). Uncropped images are reported in Figure S10.

To further verify that the observed activity was due to direct inhibition of Pin1 *in vivo*, we analyzed tumor tissues to measure **164B8** levels and Pin1 degradation in both tumor and normal tissues. At day 14, 6 h after the final intraperitoneal dose, **164B8** was detected and quantified in tumor tissue via liquid chromatography-mass spectrometry (LC-MS). Significant levels of **164B8** were detected in the liver, tumor-adjacent peritoneum, and tumor tissues collected from mice treated with either 30 mg/kg or 60 mg/kg (Figure 7A). The average tumor values were 0.62 ng/mg for mice treated with 30 mg/kg **164B8** and approximately 8 ng/mg for those treated with 60 mg/kg **164B8,** compared with the vehicle control (0.03 ng/mg). In mice treated with **164B8** at 60 mg/kg, we observed enrichment of **164B8** in tumor tissues compared with adjacent normal tissues (mean: 3.65 vs. 8 ng/mg, *p* = 0.035) and normal liver tissues (mean: 0.75 vs. 8 ng/mg, *p* = 0.0003). For the 30 mg/kg dose, drug levels were numerically higher in tumor tissue (0.62 ng/mg) compared with adjacent normal peritoneum (0.11 ng/mg) and liver (0.10 ng/mg), although these differences were not statistically significant after adjustment for multiple comparisons (Figures 7A and 7B). Likewise, Pin1 levels in tumor tissues were detected via western blotting (Figure 7C). Compared with untreated mice, tumor tissues from mice receiving **164B8** displayed significantly reduced Pin1 expression, with a reduction of approximately 30%–50%— consistent with the results of the cell-based assay reported in Figure 3, which examined the time-dependent effect of **164B8** on Pin1 degradation in cancer cells.

Collectively, these results demonstrate that **164B8** is a novel Pin1-targeting agent that effectively induces Pin1 degradation both in cell and *in vivo* (in tumor and surrounding tissues) and contributes to a significant reduction in peritoneal metastases in our mouse model of metastatic pancreatic cancer.

## Discussion and conclusions

Resistance to treatment in PDAC is attributable to the highly desmoplastic and immunosuppressive tumor microenvironment.^17^^,^^18^^,^^19^ In this context, Pin1 overexpression appears to be a major contributor, as it is highly expressed in both tumor cells and CAFs,^20^^,^^21^ prompting the questions of whether potential therapeutic options could include inhibition of Pin1 in both tumor cells and CAFs.^21^ In recent years, several such inhibitors have been proposed, including small-molecule inhibitors,^22^^,^^23^^,^^24^^,^^25^ peptide mimetics,^26^^,^^27^^,^^28^ and covalent agents targeting the active-site Cys113.^29^^,^^30^ More recently, even a PROTAC agent, which is a bi-functional molecule that simultaneously targets Pin1 (via a sulfopin-like compound) and an E3 ligase, has been reported to more effectively induce Pin1 degradation in cell.^31^ However, none of these agents have yet advanced to clinical studies, likely because they lack the general requisites for effective Pin1 targeting *in vivo*. In this context, we recently reported a novel class of Pin1 degraders that bind covalently to Cys113 and are designed to cause Pin1 destabilization *in vitro* and in cell.^32^ Here, we report on an optimized agent from this series, namely **164B8,** which induces thermal destabilization of Pin1 *in vitro*,^32^ and possesses suitable pharmacological properties to cause Pin1 degradation *in vivo*. This is attributed to the increased inhibition of Pin1 *in vitro*, greater plasma stability, and likely improved cell permeability of the agent compared with our previous compounds in the same series, resulting in more effective cellular and *in vivo* degradation of Pin1. Our studies support the notion that our Pin1 degraders could be developed into novel and effective treatments for PDAC and other gastrointestinal and abdominal cancers. The efficacy and toxicity of **164B8** were assessed in a murine model of metastatic pancreatic cancer, in which peritoneal metastases were established and mice treated with two doses of **164B8** daily for 2 weeks. Tumor burden, assessed via luminescence imaging, determined that cancer growth and peritoneal metastases were both significantly reduced in treated mice compared with the untreated cohort, in a dose-response manner. Furthermore, Pin1 and **164B8** levels were assessed in tumor and adjacent organs, indicating a direct correlation between **164B8** concentrations and Pin1 levels *in vivo*, thus providing direct evidence of Pin1 targeting by **164B8** and the associated reduction in tumor burden.

Taken together, our results corroborate the central hypothesis that Pin1 degraders with improved pharmacological properties could be developed to suppress peritoneal metastases in metastatic pancreatic cancer and potentially in other gastrointestinal and abdominal cancers. These preliminary studies encourage us to further investigate this innovative series of Pin1 degraders in other models to support their advancement into clinical studies.

## Materials and methods

### General chemistry

The solvents and reagents used for the synthesis and characterization of the agents were commercially available. All intermediate compounds and final products were purified using RP-HPLC (JASCO), equipped with a ChromNAV system and a PDA detector, and an XTerra column C18 column (10 μm 10 × 250 mm^2^; Waters). The purity of **164B8** was also determined by HPLC (Figure S3). To confirm the identity of the tested agents and correct complex adducts, high-resolution mass spectroscopy analyses were performed using an Agilent 6545 Q-TOF LC-MS instrument (Figure S5 and Table S1). Moreover, several NMR experiments were performed using a Bruker Avance III 700 MHz spectrometer equipped with a TCI cryoprobe. NMR spectra were recorded in d6- dimethyl sulfoxide (DMSO) to determine the concentration of stock solutions and in the assay buffer to ensure the solubility of the compounds used in all studies.

### **164B8** synthesis

Solid-phase peptide synthesis: **164B8** was synthesized using BAL resin, as reported in Figure S3. After swelling the resin (9 mL dichloromethane [DCM], 1 mL N-methylpyrrolidone , 15 min), loading of methylamine onto the BAL resin was carried out by adding three equivalents of amine (dissolved in 1 mL of dimethylformamide [DMF]) to the solid support and shaking for 30 min. Subsequently, three equivalents of NaBH(OAc)_3_ were added to the solution, which was then shaken overnight at room temperature.

After three washing steps with DMF and DCM, each Fmoc-amino acid (Fmoc-AA) was added using a solid-phase Fmoc peptide synthesis protocol, as described previously.^32^ Briefly, each coupling reaction was performed using three equivalents of Fmoc-AA in 1 mL of coupling solution (three equivalents of HATU [1-[Bis(dimethylamino)methylene]-1H-1,2,3-triazolo[4,5-b]pyridinium 3-oxid hexafluorophosphate]; three equivalents of Oxima Pure [ethyl cyanoglyoxylate-2-oxime]; and five equivalents of N,N-diisopropylethylamine [DIPEA] in 1 mL DMF). The reaction mixture was shaken for 2 h at room temperature. After that, the Fmoc protecting group was removed to allow coupling of the subsequent Fmoc-AA. The Fmoc deprotection solution consisted of 20% piperidine in DMF (1 mL for 5 min, followed by 1 mL for 20 min). After the peptide chain was fully synthesized, it was cleaved from the solid support using a cleavage solution composed of TFA, TIS, and water in a ratio of 94:3:3 for 3 h. Following cleavage, the solution was filtered and evaporated under reduced pressure. The amino acids utilized in the synthesis are detailed in Figure S3.

Reaction with 2-chloroacetyl chloride: The purified peptide (1 equivalent) was dissolved in DMF and shaken at room temperature. Subsequently, three equivalents of DIPEA were added to the solution, followed by three equivalents of 2-chloroacetyl chloride, which was added dropwise. The reaction mixture was stirred for 16 h (overnight) at room temperature.

### Compound purification

The purification of the tested agents was performed by RP-HPLC using a linear gradient (5 → 100% ACN (acetonitrile)/H_2_O + 0.1% TFA; duration = 30 min). The purity of **164B8** was greater than 95% (Figure S3).

### Protein expression and purification

The expression and purification of Pin1 were performed as reported recently.^32^ Residues 1–163 of human Pin1 were expressed with a thrombin cleavage site and a HisTag at the N-terminus. The 6His-Pin1 construct was expressed in LB medium to obtain the unlabeled protein and in M9 medium to obtain a uniformly ^15^N-labeled protein (kanamycin, 1 mM IPTG, 25°C, overnight). The protein was purified using nickel chromatography and eluted with a solution containing imidazole (elution buffer: 25 mM Tris, pH 7.5; 500 mM NaCl; and 500 mM imidazole). It was further purified via size-exclusion chromatography (HiLoad 26/60 Superdex 75 preparative-grade column) and exchanged into the final buffer (50 mM phosphate at pH 7.5, 150 mM NaCl, and 1 mM dithiothreitol).

### NMR spectroscopy

Heteronuclear 2D [^15^N,^1^H] correlation spectra of ^15^N-Pin1 (50 μM) in complex with **164B8** (250 μM) were collected using a Bruker Avance III 700 MHz spectrometer equipped with a TCI cryoprobe, and the data were processed with TOPSPIN 4.1.0 (Bruker, Billerica, MA). 2D-[^15^N,^1^H]-SOFAST-HSQC experiments were acquired using 64 scans per increment with 2048 and 128 complex data points in the ^1^H and ^15^N dimensions, respectively, at 298 K (Figure S4).

### Denaturation thermal shift measurements

Thermal shift assays for Pin1 were conducted as described previously,^32^ using a Bio-Rad CFX Connect Real-Time PCR detection system and SYPRO Orange. Pin1 samples (in 50 mM phosphate buffer, pH 7.5, 150 mM NaCl, 3% DMSO) were tested in the absence and presence of compounds (protein:ligand ratio 1:2, 20 μM protein), and ΔTm values were collected in quadruplicate. For the denaturation curves, the temperature was increased from 10°C to 95°C in 0.05°C increments over 30 min.

### DELFIA

The assay was carried out as we recently described,^32^ using His-tagged-Pin1 and an Eu-N1-labeled anti-6×-His antibody (PerkinElmer, 1:2000), along with a biotinylated D-peptide bound to streptavidin-coated plates.^32^

### Cell culture

BxPC3 and MIA PaCa-2 were obtained from the American Type Culture Collection, and KPC cells were a kind gift from Dr. Neil Bhowmick’s laboratory (Cedars-Sinai Medical Center). BxPC3 cells were cultured in RPMI (Corning), whereas MIA PaCa-2 and KPC cells were cultured in DMEM (Corning). All culture media were supplemented with 10% FBS, and 2.5% horse serum (Gibco) was added only to MIA PaCa-2 cells. Cells were maintained in a humidified incubator at 37°C with 5% CO_2_.

### Immunoblotting

At the end of the treatments, cells were lysed with a lysis buffer containing 20 mM Tris (pH 7.4), 1% Triton X-100, 5 mM EDTA, 0.1% sodium dodecyl sulfate (SDS), 1% IGEPAL, 0.5% sodium deoxycholate, 120 mM NaCl, and a protease inhibitor cocktail and PhosSTOP (Sigma-Aldrich). Lysates were then centrifuged at 16,000 × g for 20 min at 4°C, and the supernatants were collected. Protein concentrations were determined using the Pierce BCA Protein Assay Kit (Thermo Fisher Scientific) according to the manufacturer’s protocol. For traditional western blotting (Figure 2), samples were loaded onto precast Bis-Tris gels and subsequently transferred onto PVDF membranes. Blots were blocked with 5% nonfat milk for 1 h and incubated with anti-Pin1 (Cell Signaling Technology, #3722) or anti-β-actin (Santa Cruz Biotechnology, #sc-69879) antibodies overnight at 4°C. Blots were then incubated with appropriate HRP-conjugated secondary antibodies for 1 h at room temperature, and Clarity Western ECL solution (Bio-Rad) was added. Imaging was carried out using a ChemiDoc imaging system (Bio-Rad). Uncropped images of western blots are available in Figure S6.

For capillary electrophoresis immunoassays (Figure 3), following protein quantification via the bicinchoninic acid (BCA) assay, the Jess Simple Western instrument (Protein Simple, Bio-Techne, Minneapolis, MN, USA) was utilized according to the manufacturer’s protocol for the 12–230 kDa separation module (SM-W001, Bio-Techne). Briefly, cell lysates were run at a concentration of 1.75 mg/mL after dilution in 1× fluorescent master mix. A total of 3 μL of lysate was loaded per well, and 1:20 anti-β-actin (Santa Cruz Biotechnology, #sc-69879) and 1:250 anti-Pin1 (Cell Signaling Technology, #3722) antibodies were used. Primary antibodies were diluted in antibody diluent 2 (042–203, Bio-Techne), and secondary antibodies were added according to the manufacturer’s protocol (DM-001 and DM-002, Bio-Techne). The resulting data were analyzed using Compass for Simple Western software (v.6.3.0), utilizing the default high dynamic range 4.0 image settings and Gaussian peak fitting to obtain the area under each curve for quantification and analysis. Duplicate experiments and uncropped images of the capillaries from the Jess instrument are shown in Figure S7**.**

### CAFs and normal peritoneal fibroblasts

Primary human CAFs and normal peritoneal fibroblasts were isolated at the City of Hope Medical Center from freshly resected gastrointestinal tumors, including cholangiocarcinoma (biliary tract), appendiceal carcinoma, and colorectal cancer peritoneal metastases (COH IRBs 18209, 19184, 21679), as well as from normal peritoneal tissue, using a standard tissue outgrowth method. The resulting fibroblast populations were validated as CAFs based on their spindle-shaped morphology and flow cytometric staining—positive for α-smooth muscle actin and CD90, and negative for epithelial cell adhesion molecule. Only early-passage cultures (≤ passage 10) were used for experiments. The cell lines were cultured in Dulbecco’s Modified Eagle Medium/Ham’s F-12 (Gibco), supplemented with 10% fetal bovine serum, penicillin-streptomycin (100 U/mL), HEPES (10 mM), and GlutaMAX HEPES (2 mM). Cell cultures were maintained at 37°C in a humidified atmosphere with 5% CO_2_. Uncropped western blot images are shown in Figure S8.

### Determination of Pin1 levels in tumor tissues

Tumor tissues were freshly harvested from mice in RPMI and immediately placed on ice. For protein extraction, tissues were first weighed and then homogenized at a ratio of 50 mg tissue per 500 μL of ice-cold lysis buffer (50 mM Tris-HCl, pH 7.5, 150 mM NaCl, 1% NP-40, 0.5% sodium deoxycholate, and 0.1% SDS), freshly supplemented with protease and phosphatase inhibitor cocktails (Roche). The tissues were finely minced on ice, sonicated (30% amplitude, 5 s on, 10 s off; total 1 min), and centrifuged at 14,000 × g for 15 min at 4°C. The supernatants were collected, and protein concentration was determined using the BCA protein assay kit (Thermo Fisher Scientific). Equal amounts of protein were analyzed by traditional western blotting, as described below.

### Molecular modeling

The model of **164B8** in complex with Pin1 was prepared based on our previously determined X-ray structure of compounds from this series in complex with Pin1 (PDB ID 8VJF) using Sybyl-X. The model coordinates are provided in the supplemental information. Figures including molecular models were prepared using ChimeraX v.1.7.^34^

### Aqueous and plasma stability studies

A solution of a Pin1 inhibitor was spiked into blank plasma at 1 μg/mL in various solutions, including an aqueous buffer (1× phosphate-buffered saline; Fisher Scientific, Fair Lawn, NJ), human plasma (Innovative Research Inc., Novi, MI), wild-type mouse plasma (Innovative Research Inc., Novi, MI), plasma form esterase-deficient mice (City of Hope, Duarte, CA), and plasma from Black 6-Cg mice (City of Hope, Duarte, CA).

The spiked plasma samples were incubated at 37°C for up to 6 h. At each designated time point, a 20 μL aliquot of the incubation sample was removed and quenched in 100 μL of acetonitrile. At the end of the incubation, the quenched samples were vortexed and centrifuged for 10 min at 4°C and 20,000 × g. The resulting supernatant was reconstituted and analyzed by LC-MS/MS.

### *In vivo* xenograft studies

Male NSG mice, approximately 20 weeks old, obtained from the Animal Resources Center of the City of Hope, were used for the xenograft study according to the City of Hope IACUC approved protocols. KPC tumor cells transfected with luciferase were injected intraperitoneally at 200,000 cells in 100 μL. KPC cells were transduced with a lentiviral vector encoding a luciferase-eGFP fusion protein. Lentiviral particles were generated by transfection of HEK293T cells with the transfer plasmid, psPAX2 packaging plasmid, and pMD2.G envelope plasmid using lipofectamine 3000 (Thermo Fisher Scientific). Viral supernatants were collected at 48 h post-transfection, filtered through a 0.45 μm filter, and applied to KPC cells in the presence of 8 μg/mL polybrene. 72 h post-infection, GFP expression was verified by fluorescence microscopy, and luciferase activity was confirmed using a Cytation3 reader.

Bioluminescent imaging (BLI) was performed one week post-inoculation. Based on imaging intensity, mice were re-grouped to ensure six animals per group. The PIN1 inhibitor was administered via intraperitoneal injection at doses of 30 mg/kg or 60 mg/kg, once daily, 5 days per week for 2 weeks. Tumor engraftment and growth were monitored by *in vivo* BLI. D-luciferin potassium salt was dissolved in sterile PBS to a final concentration of 16.6 mg/mL and mice were injected intraperitoneally with D-luciferin solution (0.139 g luciferin/kg body weight), then imaged 10–15 min after luciferin injection using an IVIS Spectrum Imaging System (Lago X) with identical exposure times and binning settings for all mice. Bioluminescence signal intensity was quantified as total flux (photons/s) within a region of interest (ROI) using Aura image software. BLI was performed once every week during the treatment. After 2 weeks of treatment, mice were euthanized 6 h after the final dose. Blood samples were collected via cardiac puncture, and normal peritoneum, intestine, and liver tissues were harvested and snap-frozen on dry ice.

## Data and code availability

All data are available as reported in the manuscript and in the supplemental information.

## Acknowledgments

Financial support was obtained in part from 10.13039/100000002NIH grants NS107479 (to M.P.), CA168517 (to M.P.), and CA242620 and CA285114 (to M.P. and M.R.). M.P. holds the Daniel Hays Chair in Cancer Research at the School of Medicine at UCR. Some molecular graphics and analyses were performed using UCSF Chimera. Chimera was developed by the Resource for Biocomputing, Visualization, and Informatics at the 10.13039/100008069University of California, San Francisco, with support from NIH grant P41-GM103311.

## Author contributions

G.A. and T.L., under the supervision and guidance of M.P. and M.R., respectively, contributed to the research design and conducted several of the reported studies. Specifically, G.A. designed and synthesized the agents, including control compounds and their characterizations, and performed all biochemical and biophysical studies reported. T.L. conducted most of the cellular assays reported and, under the supervision of J.W. and M.R., and with the help of F.X., contributed to the *in vivo* studies. A.M.P. conducted the capillary electrophoresis studies. P.U. conducted the western blots studies in pancreatic cancer cell lines. M.F., under the supervision of T.S., conducted the plasma stability and toxicity studies. M.P. and M.R. directed the studies and analyzed all data from their respective laboratories. M.P., with G.A., wrote the manuscript with the help of M.R. and contributions from all authors.

## Declaration of interests

M.P. is a co-founder of Armida Labs, Inc. (Riverside, CA, USA). M.P., P.U., and G.A. are listed as possible co-inventors on a patent application filed by UCR related to the compounds described in the application.

## Supplemental information

Document S1. Figures S1–S14 and Tables S1 and S2

Document S2. Article plus supplemental information
