# Supplementary material for: Pre-clinical evaluation of a potent and effective Pin1-degrading agent in pancreatic cancer
Source: Mol Ther Oncol. 2025 Nov 1;33(4):201078. doi: 10.1016/j.omton.2025.201078 (PMC12657321; doi:10.1016/j.omton.2025.201078)
Supplement: Document S1. Figures S1–S14 and Tables S1 and S2 [file mmc1.pdf]

OMTON, Volume *33*

Supplemental information

Pre-clinical evaluation of a potent and effective Pin1-degrading agent in pancreatic cancer

Giulia Alboreggia, Tiane Li, Anne Marie Prentiss, Parima Udompholkul, Frank Xia, Tim Synold, Jun Wu, Mingye Feng, Mustafa Raoof, and Maurizio Pellecchia

D-peptide

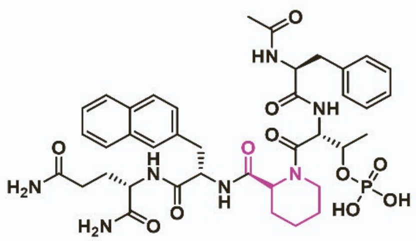

164B8

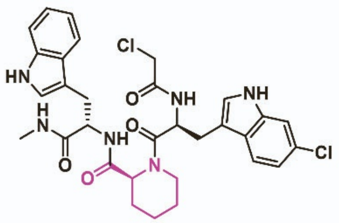

Cmpd	MW	IC_{50} (nM)	ΔT_m (°C)
D-peptide	823.33	2912 ± 103	3.40 ± 0.16
164B8	625	4.65 ± 0.04	-9.55 ± 0.19

Figure S1: comparison of D-peptide and **164B8.** Chemical structures and characterizations of Pin1 inhibitors D-peptide and **164B8**. IC_{50} values were obtained by dose-response curves in DELFIA displacement assay after compound-ligand incubation time of 6 h. Standard errors reported are the results of duplicate measurements. ΔTm values were measured in quadruplicates as described in the manuscript.

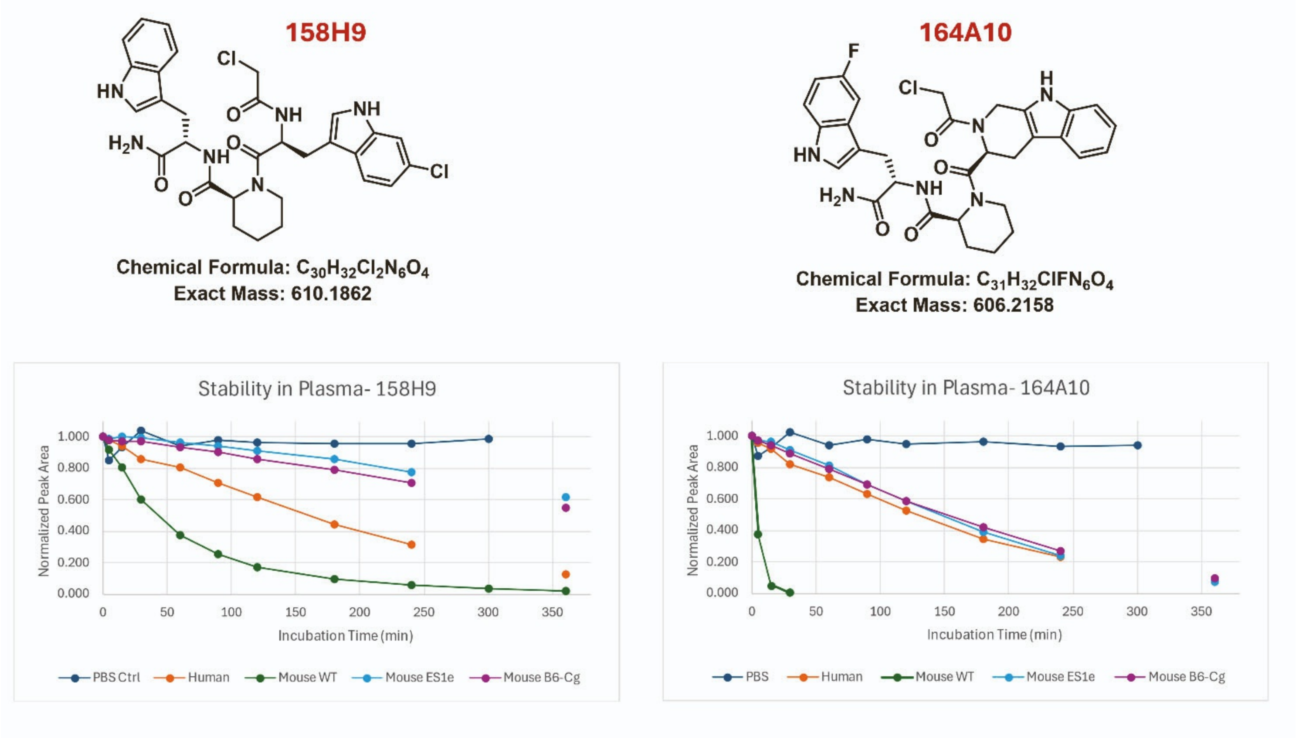

Figure S2: Stability half-life of agents **158H9** and **164A10** after incubation at 37 ºC in various media. *Acq* represents aqueous buffer composed of 1x phosphate buffered saline (Fisher Scientific, Fair Lawn, NJ); *human* represents human plasma (Innovative Research Inc., Novi, MI); *mouse wt* represents wild type mouse plasma (Innovative Research Inc., Novi, MI); *mouse ES1e* represents plasma form esterase deficient mice (City of Hope, Duarte, CA); *mouse B6-Cg* represents plasma from black 6-Cg mice (City of Hope, Duarte, CA).

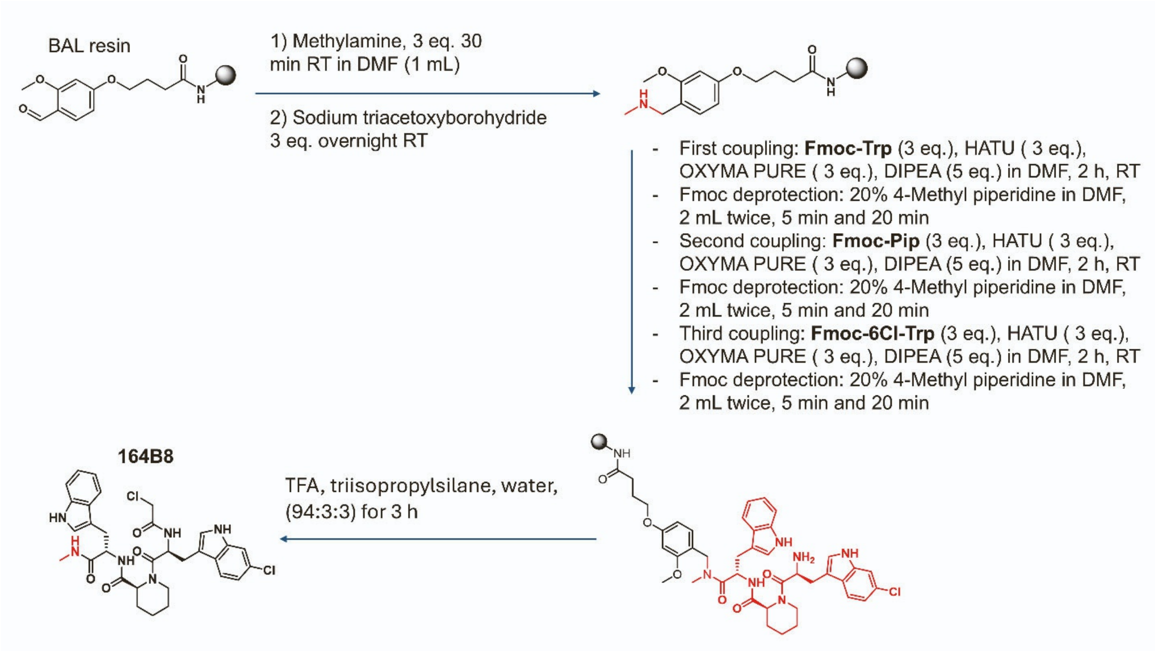

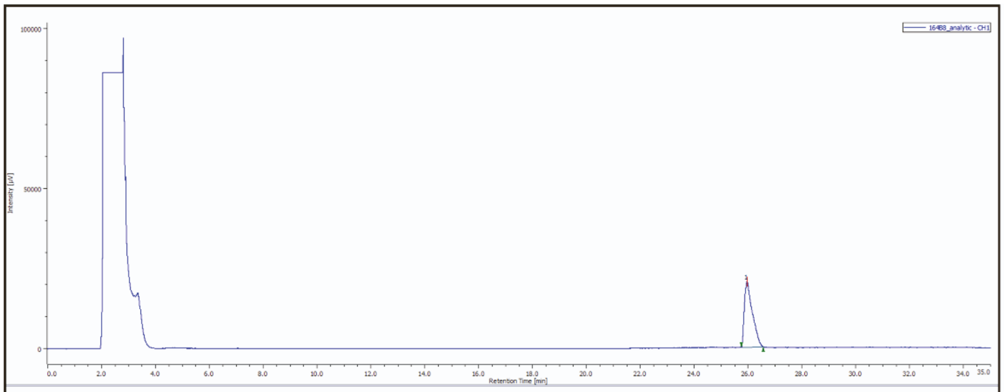

#	Peak Name	CH	tR	Area	Height	Area%	Height%	Quantity	NTP	Resolution	Symmetry Factor	Warning
1	Unknown	1	25.950	418081	20401	100.000	100.000	N/A	34948	N/A	1.815	

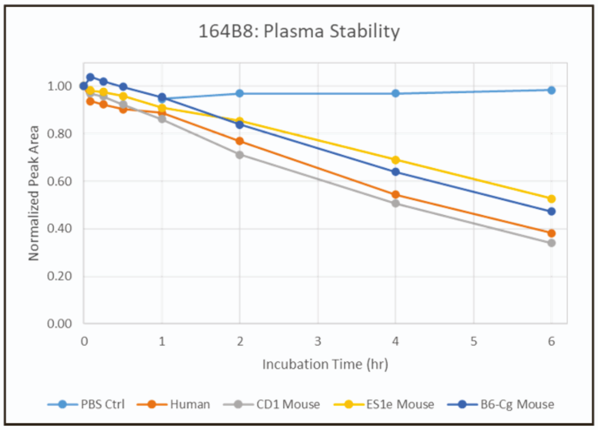

Figure S3: Synthetic route of agent **164B8**. HPLC purity (> 95%) and plasma stability over time are also reported.

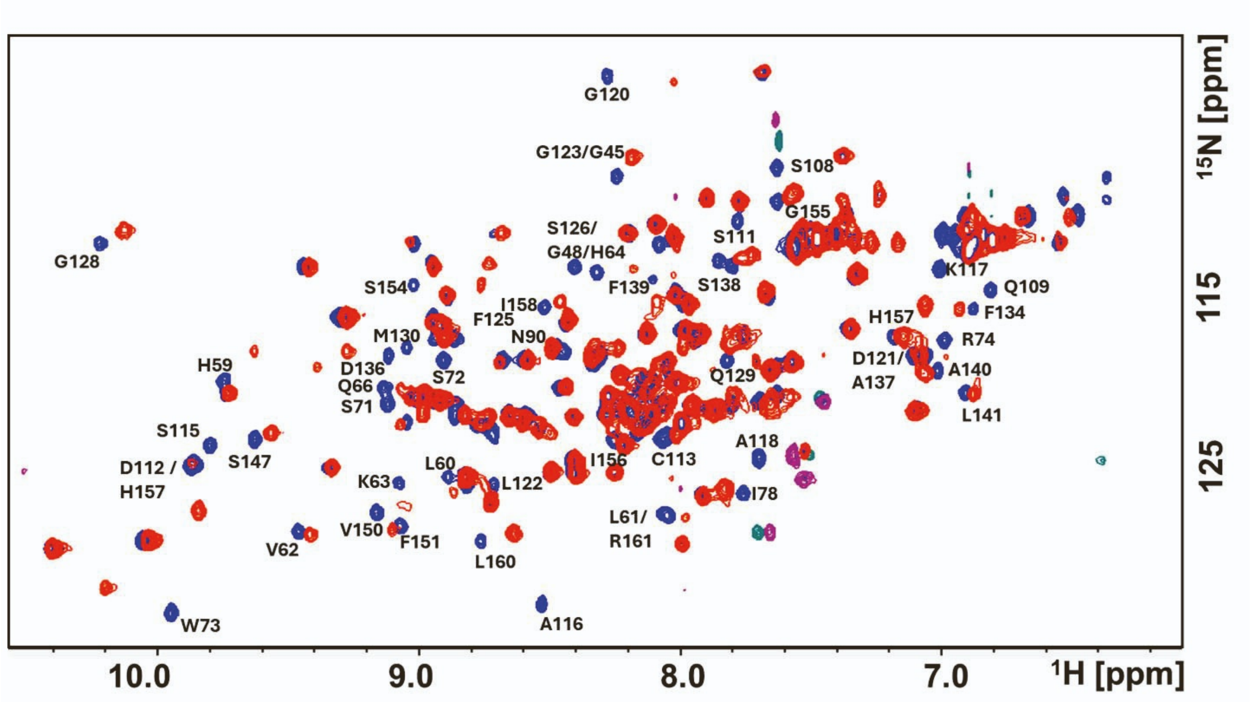

Figure S4: Backbone 2D [^{15}N,^{1}H] correlation spectra for ^{15}N-Pin1 (50 μM) collected in absence (blue) or presence of **164B8** (250 μM) after 3 h incubation. Resonance assignments are also reported.

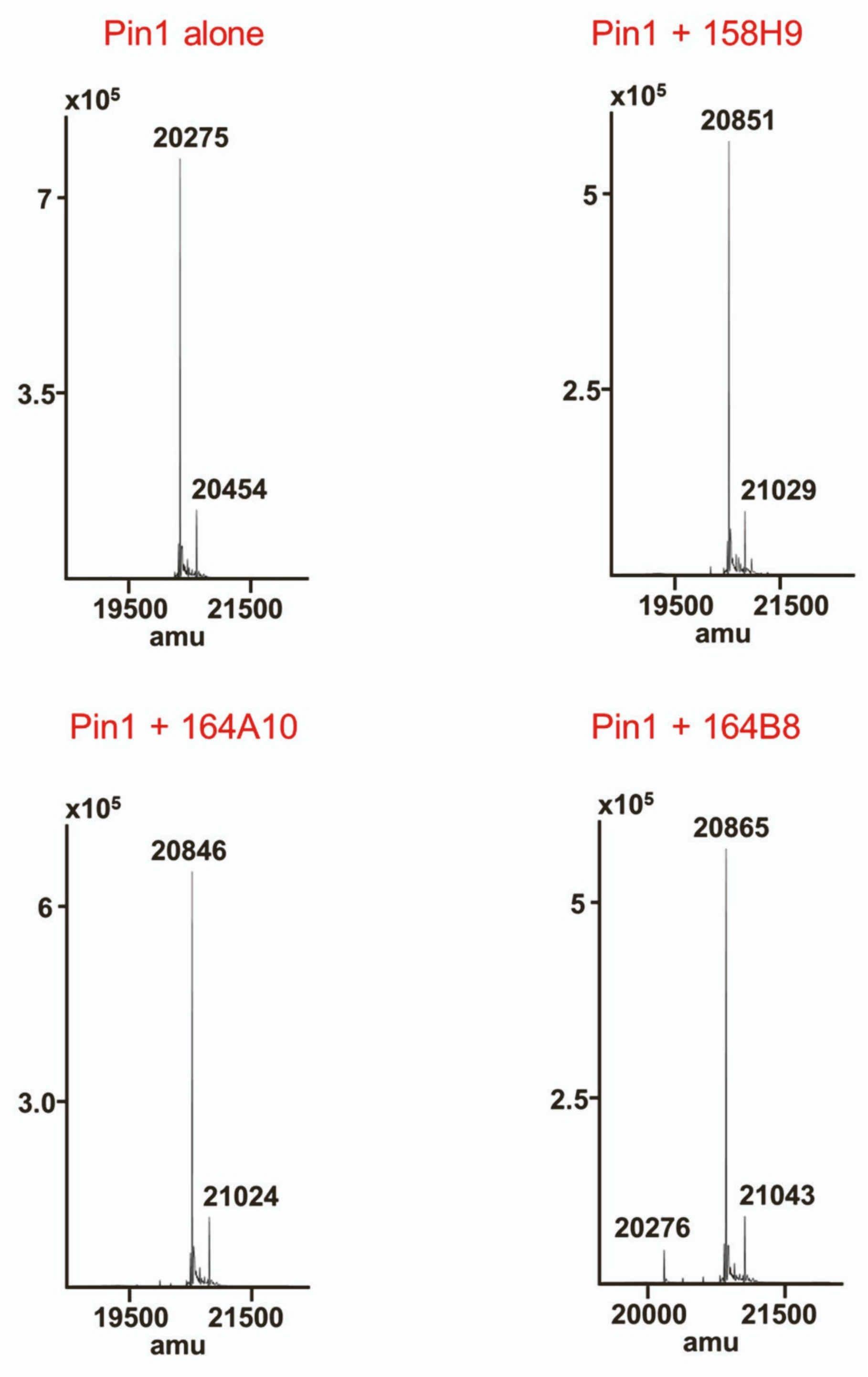

Figure S5: Mass spectrometry analyses of Pin1 (10 μM) collected in presence of **158H9**, **164A10**, or **164B8** (compounds 20 μM, 10 minutes incubation, rt).

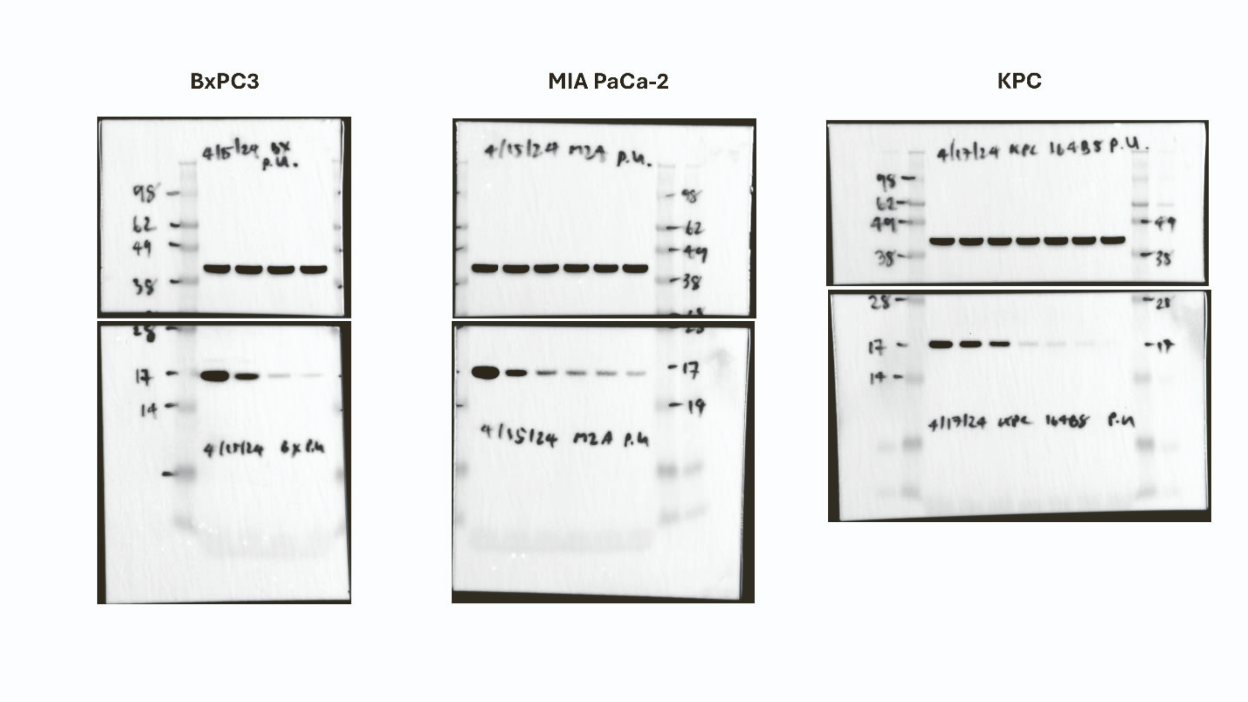

Figure S6: Uncropped western blot images relative to the data reported in **Figure 2**.

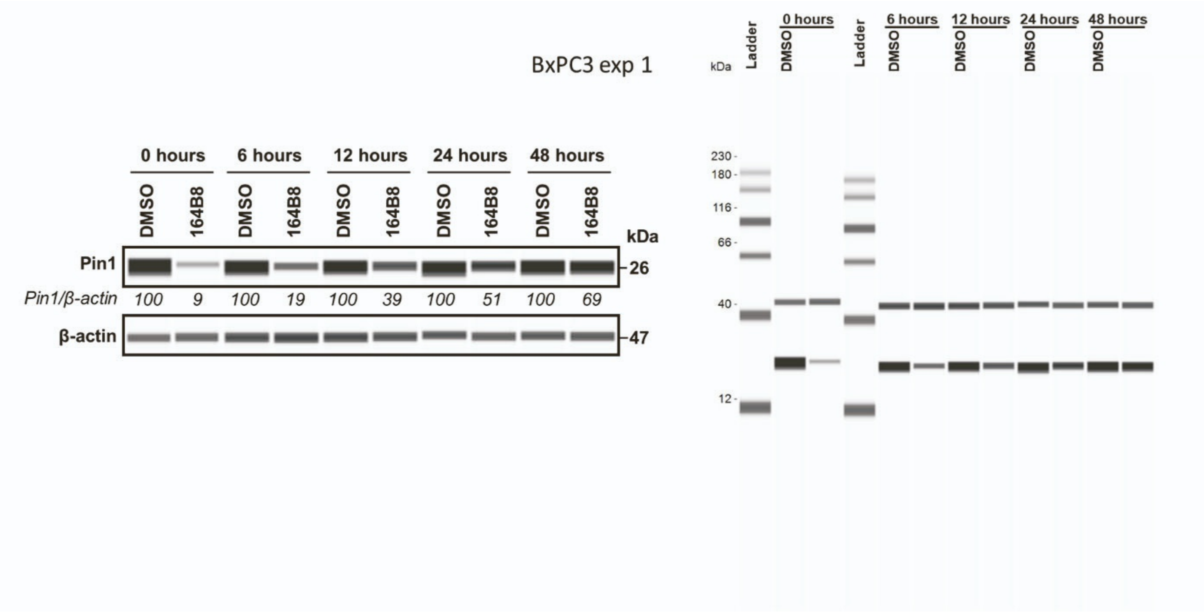
BxPC3 exp 1
0 hours
6 hours
12 hours
24 hours
48 hours
DMSO
164B8
kDa
Pin1
26
Pin1/β-actin
100 9 100 19 100 39 100 51 100 69
β-actin
47
Ladder
230
180
116
66
40
12

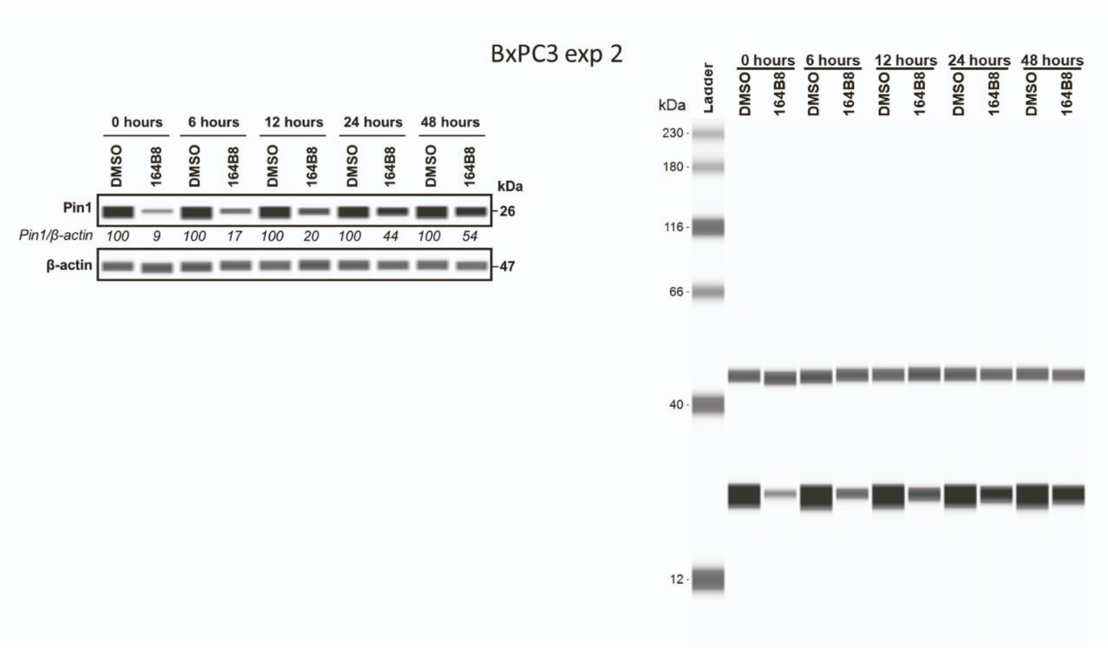
BxPC3 exp 2
0 hours
6 hours
12 hours
24 hours
48 hours
DMSO
164B8
kDa
Pin1
26
Pin1/β-actin
100 9 100 17 100 20 100 44 100 54
β-actin
47
Ladder
230
180
116
66
40
12

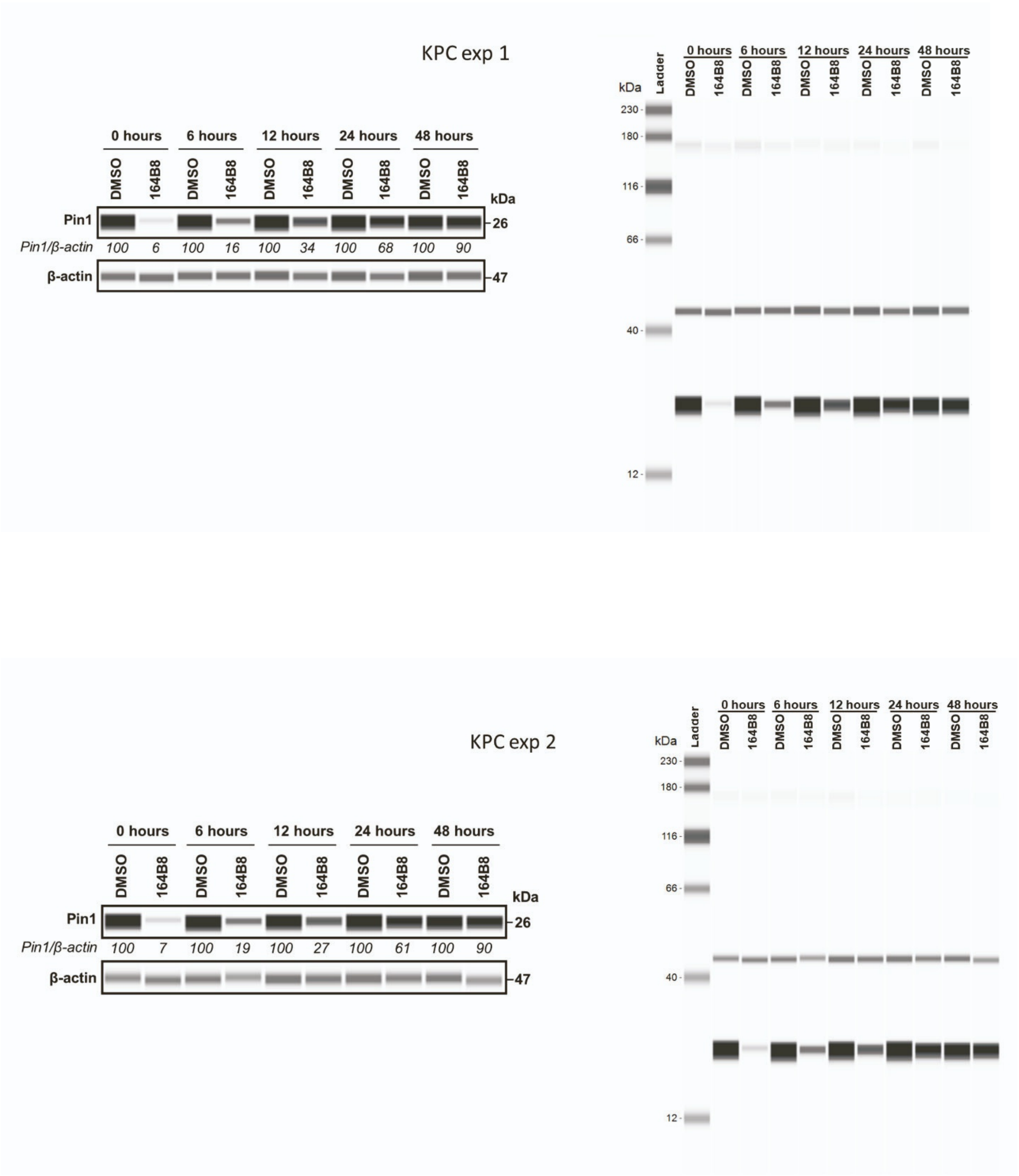

Figure S7: Results and uncropped images of the duplicate capillary electrophoresis experiments relative to **Figure 3**.

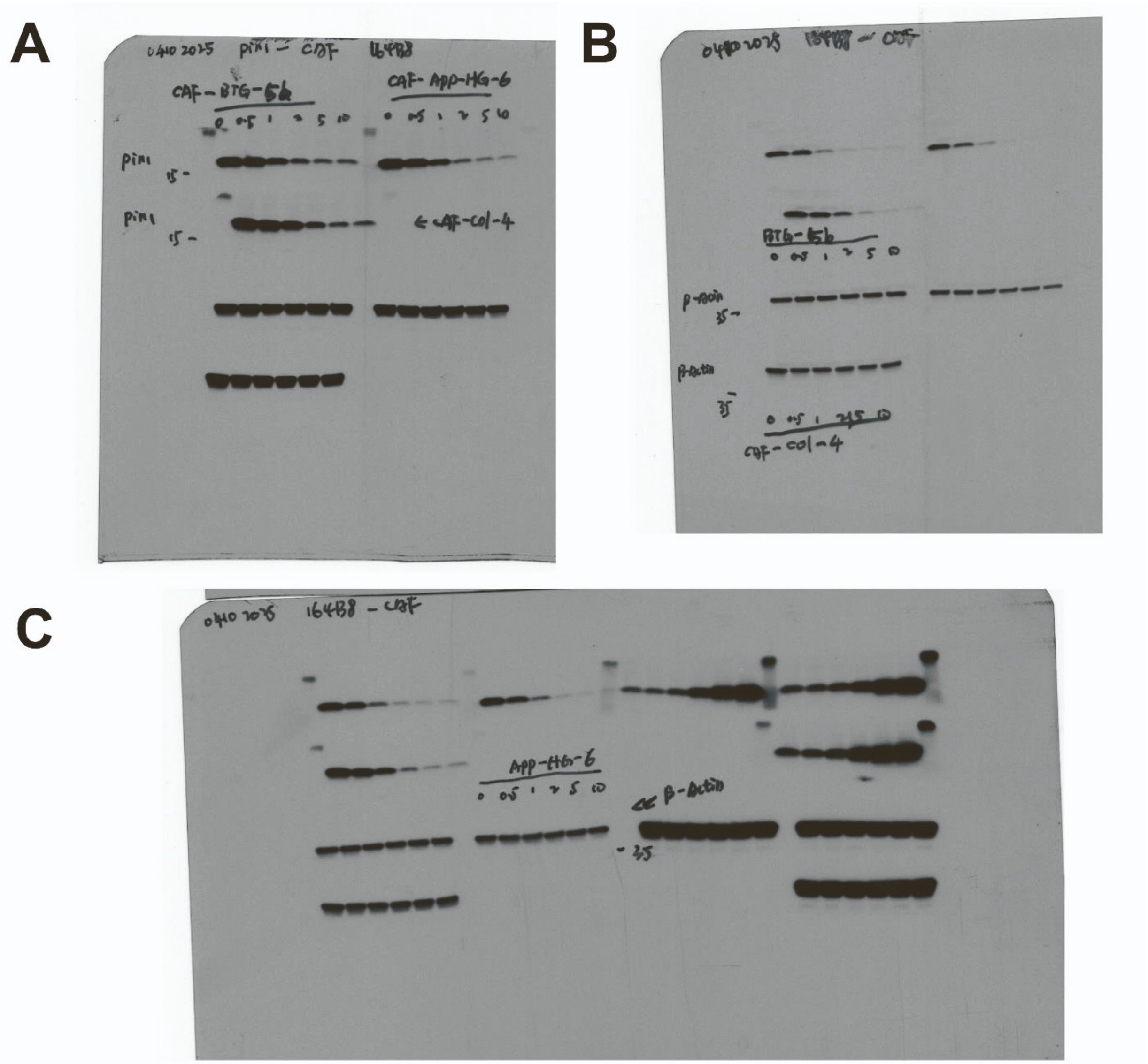

Figure S8: Uncropped Western Blot images (data reported in **Figure 4**). Pin1 bands presented are reported in Panel A. β-Actin bands reported for CAF-BTG and CAF-COL are reported in Panel B. β-Actin bands reported for CAF-APP are reported in Panel C.

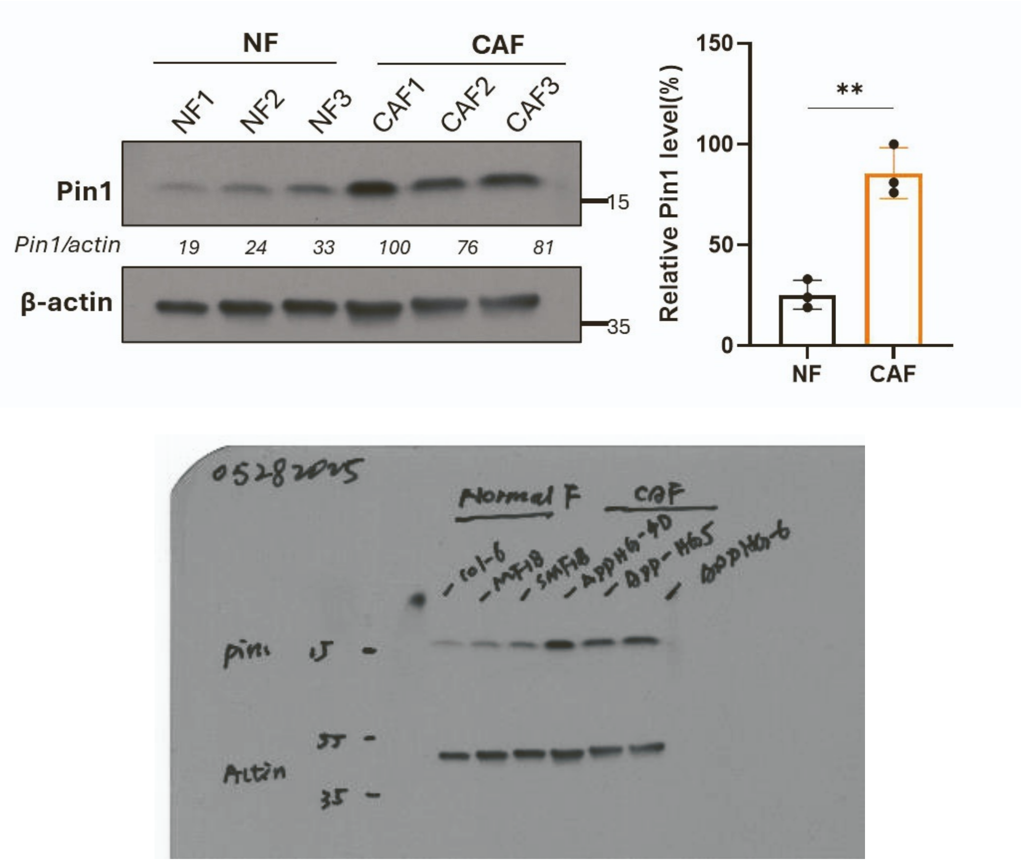

Figure S9: Pin1 protein level in normal peritoneal fibroblasts (NFs) and primary cancer-associated fibroblasts (CAFs). CAFs and NFs were isolated from patient-derived appendiceal cancer peritoneal metastases tissue and normal peritoneal tissue. A total of three independent NF lines and three CAF lines were analyzed. Pin1 protein levels were assessed by immunoblotting using a specific anti-Pin1 antibody, with β-actin serving as the loading control.

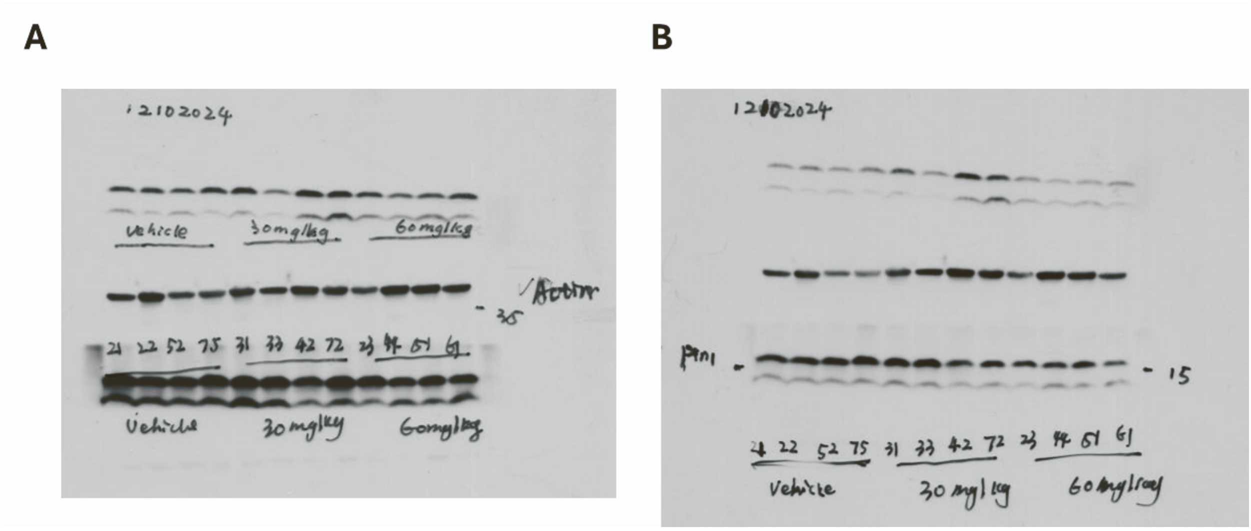

Figure S10: Uncropped Western Blot images (relative to **Figure 7**). β-Actin bands are reported in Panel A. Pin1 bands are reported in Panel B.

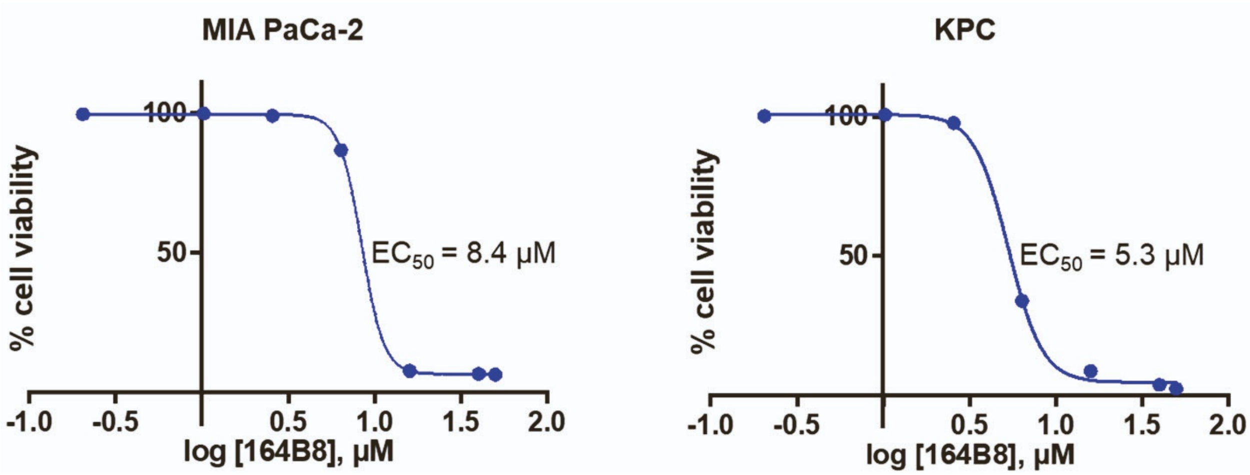

Figure S11: Mia PaCa-2 and KPC cells were treated with different doses of **164B8**, and the IncuCyte S3 live-cell analysis system was used to monitor the agent's activity every 3 hours for 72 hours. The resulting EC_{50} values after 72 hours are reported as 8.4 µM in MIA PaCa-2 and 5.3 µM in KPC cell lines.

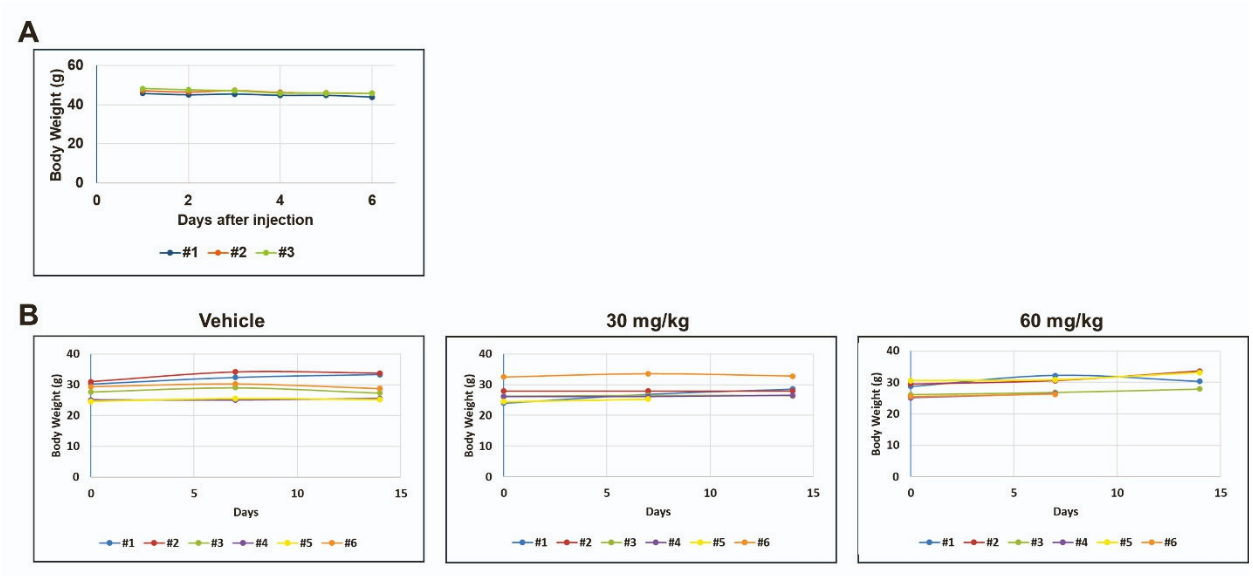

Figure S12: A) Acute toxicity study at 100mg/kg. In this preliminary safety study, 3 CD-1 mice were treated with a single dose of 100 mg/kg of **164B8**. The injections were administered intraperitoneally (ip), and the body weight of the mice was measured for several days after the injection. B) Body weight control during the in-vivo efficacy study. The body weights of the mice in the three groups were measured on the day of treatment (day 0) and 7 and 14 days after.

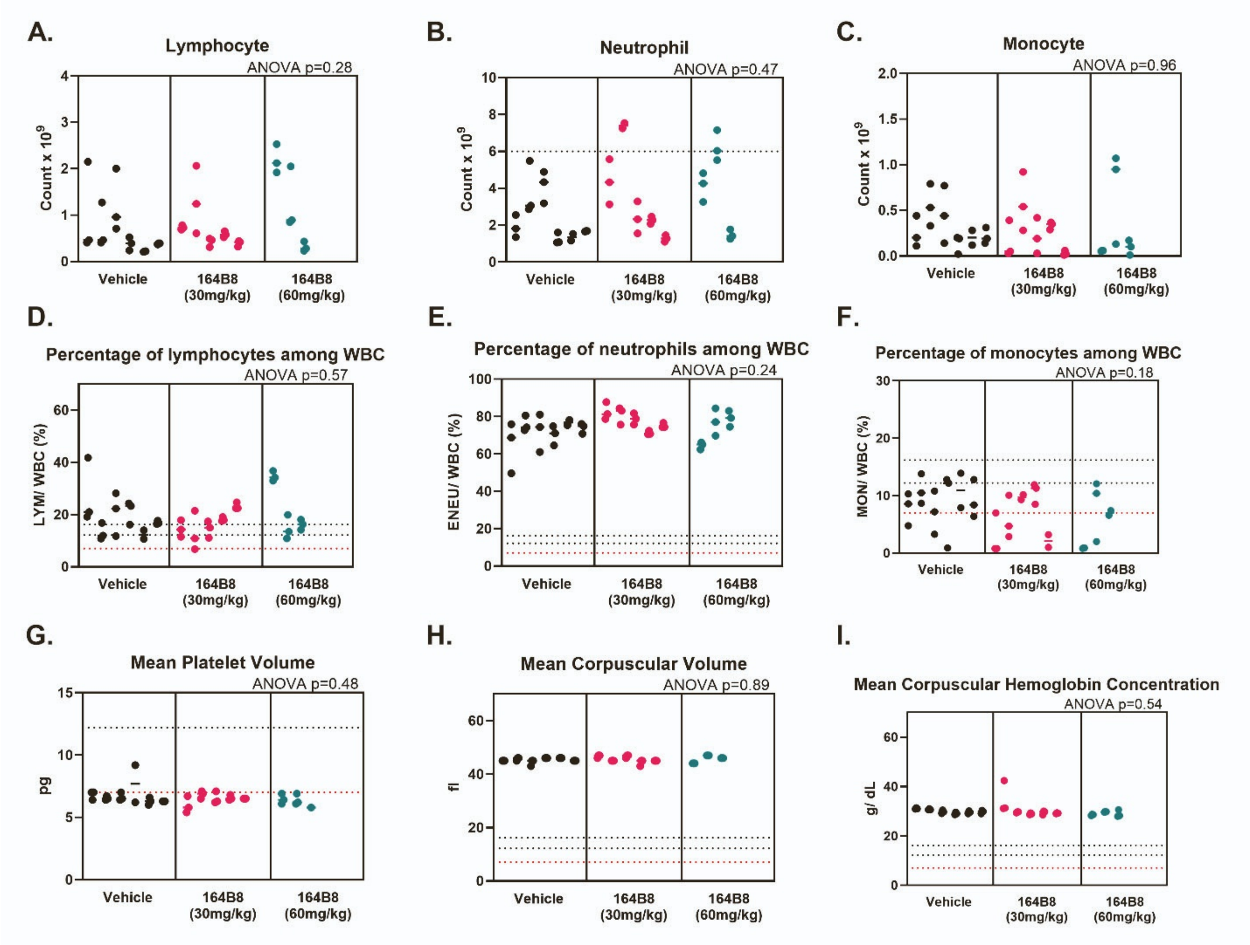

Figure S13: Complete blood count (CBC) parameters in mice after repeated **164B8** dosing. Mice were treated with vehicle or **164B8** at 30mg/kg or 60mg/kg. Peripheral blood was collected at study end from mice and analyzed by standard CBC. A) Lymphocyte counts ($\times 10^9$ cells/L). B) Neutrophil counts ($\times 10^9$ cells/L). C)Monocyte counts ($\times 10^9$ cells/L). D)Lymphocytes as a percentage of total white blood cells (WBC) (%). E) Neutrophils as a percentage of WBC (%). F) Monocytes as a percentage of WBC (%). G) Mean platelet volume (MPV, pg; picograms, 10^{-12} g), reflecting the average mass of hemoglobin per platelet and serving as an index of platelet size and activity. H) Mean corpuscular volume (MCV, fl; femtoliters, 10^{-15} L) representing the average volume of individual red blood cells and used to classify anemia as microcytic or macrocytic. I)Mean corpuscular hemoglobin concentration (MCHC, g/dL), measuring the average concentration of hemoglobin per unit volume of red blood cells, was measured by a hematology

machine. Dotted horizontal lines indicated physiological reference ranges. P values from one-way ANOVA are shown above each panel.

A

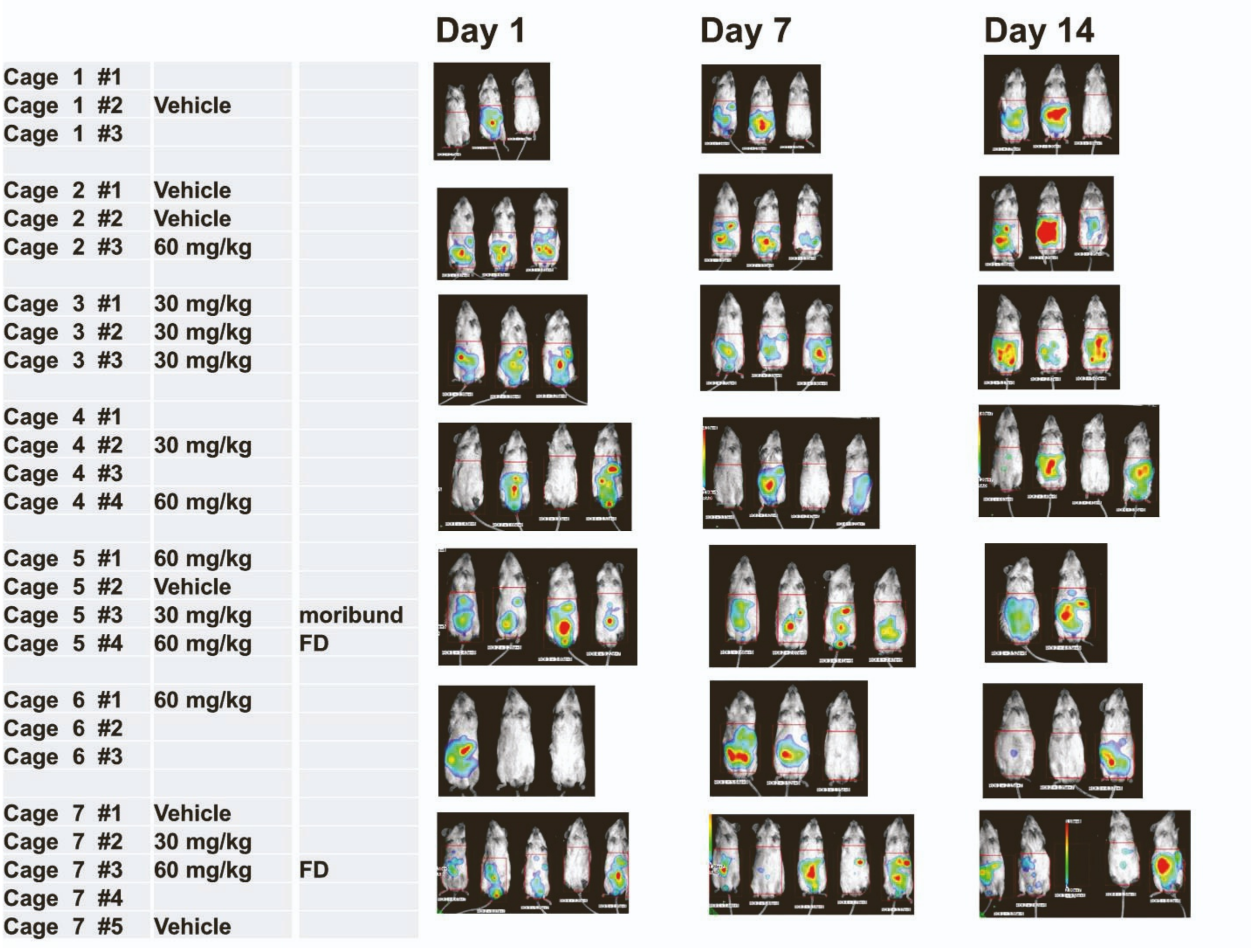

B

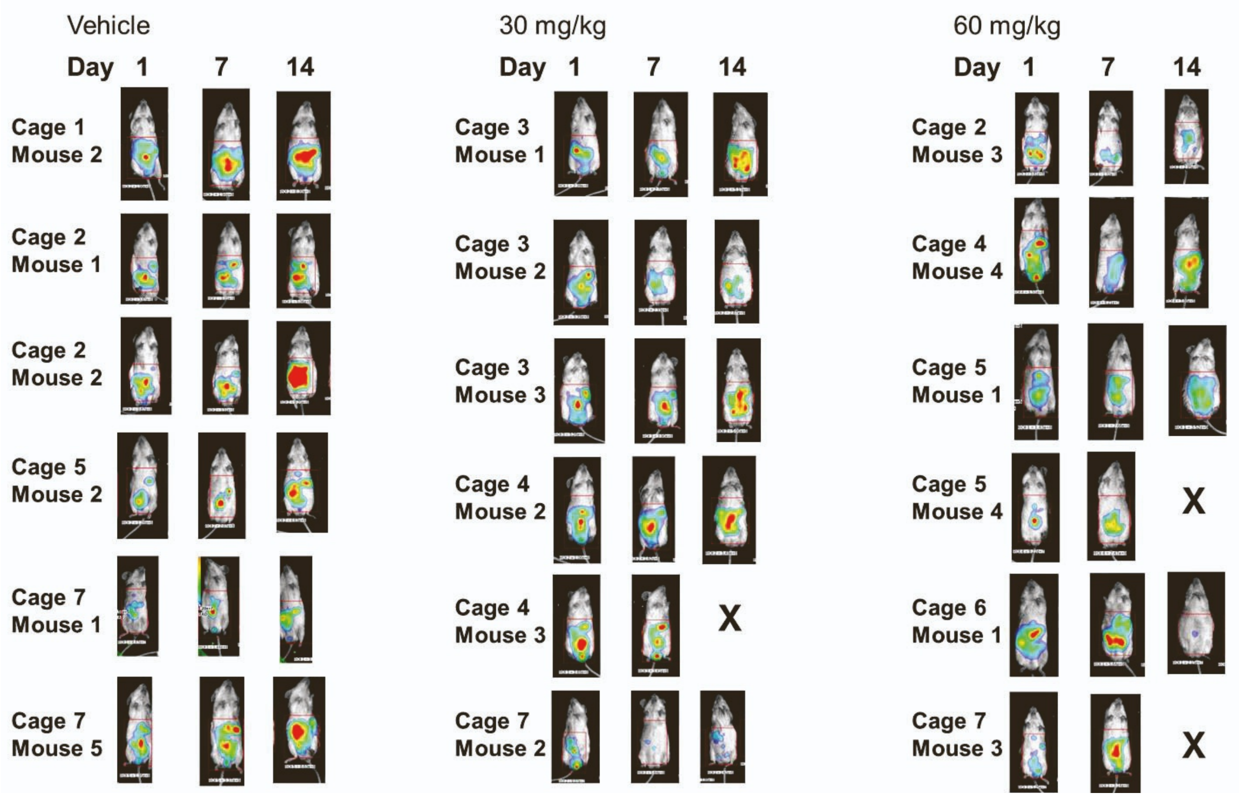

Figure S14: Images of in vivo analysis of mice. **A**) Luminescence whole body images of mice from the in vivo efficacy study. Images for all three groups, taken at days 0, 7, or 14, are presented. **B**) Reorganization of the images presented in panel A to show each mouse individually at the 3 time points.

Table S1. Mass-spectrometry data of the compounds and the adducts. All the samples were analyzed using an Agilent 6545 QTOF LC/MS instrument.

ID	Calcd [M]	Obs. (m/z)
**158H9**	610.1863	$[M+Na]^+$ = 633.1757
**164A10**	606.2158	$[M+Na]^+$ = 629.2052
**164B8**	624.2019	$[M+H]^+$ = 625.2087<br>$[M+Na]^+$ = 647.1918

ID	Calcd [M]	Obs. (m/z)
**Pin1**	20275.41	[M] = 20275<br>[M] +178 = 20454
**Pin1 + 158H9**	20849.6	$[M+H]^+$ = 20851<br>$[M+H]^+$ +178 = 21029
**Pin1 + 164A10**	20845.6	$[M+H]^+$ = 20846<br>$[M+H]^+$ +178 = 21024
**Pin1 + 164B8**	20863.6	$[M+H]^+$ = 20865<br>$[M+H]^+$ +178 = 21043

Table S2. Stability half-life of agents reported in Table 1 after incubation at 37 oC in various media. Acq represents aqueous buffer composed of 1x phosphate buffered saline (Fisher Scientific, Fair Lawn, NJ); human represents human plasma (Innovative Research Inc., Novi, MI); mouse wt represents wild type mouse plasma (Innovative Research Inc., Novi, MI); mouse ES1e represents plasma form esterase deficient mice (City of Hope, Duarte, CA); mouse B6-Cg represents plasma from black 6-Cg mice (City of Hope, Duarte, CA). See Figure 1 for the chemical structures of these compounds.

	Stability half-life				
**Compound**	***Acq***	***Human***	***Mouse WT***	***Mouse ES1e***	***Mouse B6-Cg***
**158H9**	**> 5 h**	**~ 3 h**	**~ 30 min**	**> 5 h**	**> 5 h**
**164A10**	**> 5 h**	**~ 2.5 h**	**< 10 min**	**~ 2.5 h**	**~ 2.5 h**
**164B8**	**> 5 h**	**~ 4.5 h**	**~ 4.5 h**	**> 5 h**	**> 5 h**
